# Differential approach to peripheral blood cell ratios in patients with systemic lupus erythematosus and various manifestations

**DOI:** 10.1007/s00296-020-04669-3

**Published:** 2020-08-09

**Authors:** Dorota Suszek, Anna Górak, Maria Majdan

**Affiliations:** Department of Rheumatology and Connective Tissue Diseases, The Medical University of Lublin, Ul. Jaczewskiego 8, 20-954 Lublin, Poland

**Keywords:** Lupus erythematosus, Haematological markers, Disease activity

## Abstract

New markers of systemic lupus erythematosus (SLE) activity are under investigation. In recent years, the researchers have been focusing increased attention on the role of haematological indicators in assessing the disease activity. Specifically, neutrophil-, basophil-, eosinophil-, monocyte- and platelet-to-lymphocyte ratios (NLR, BLR, ELR, MLR and PLR) have been considered. The specific objective of this study was to determine the suitability of the haematological markers for the assessment of SLE activity and SLE-related organ damage. This study is a retrospective analysis of 136 patients with SLE (124 women and 12 men) who received chloroquine/hydroxychloroquine (HQ/HCQ) monotherapy or HQ/HCQ therapy combined with low/medium doses of glucocorticoid. All patients were assessed for disease activity using the Systemic Lupus Erythematosus Disease Activity Index 2000 (SLEDAI-2K) scale. In addition, erythrocyte sedimentation rate (ESR) and C-reactive protein (CRP) inflammatory parameters were determined in each patient. NLR, BLR, ELR, MLR and PLR were evaluated and correlated with the SLE activity parameters and inflammatory markers. The mean values of the haematological indicators were compared in particular manifestations of SLE-induced organ damage. For numerical variables, descriptive statistics were calculated: median, standard deviation, minimum and maximum values. The Mann–Whitney *U* test was used for the comparison of continuous variables in the two groups. The Spearman rank correlation test was used to search for any relationships between variables. A *p* value < 0.05 was considered to be statistically significant. We have found a positive correlation between ELR, MLR and the SLEDAI scale (*r* = 0.22 and *r* = 0.27, respectively). NLR, MLR and PLR ratios were significantly correlated with ESR and CRP. Considerably higher NLR values were found in patients with cutaneous and/or mucosal symptoms and with kidney involvement compared to patients without such involvement (4.26 ± 4.2 vs 3.27 ± 2.7; *p* = 0.05 and 5.45 ± 5.6 vs 3.05 ± 2.0; *p* < 0.001 respectively). BLR and MLR were significantly higher in patients manifesting symptoms of vasculitis (0.09 ± 0.1 vs 0.02 ± 0.01; *p* < 0.001 and 3.1 ± 4.2 vs 0.3 ± 0.1; *p* < 0.001 respectively), arthritis and/or myositis (0.04 ± 0.09 vs 0.02 ± 0.01; *p* = 0.01 and 1.02 ± 2.6 vs 0.35 ± 0.4; *p* = 0.01 respectively), whereas elevated ELR ratios were observed in patients with vasculitis (0.4 ± 0.5 vs 0.08 ± 0.06; *p* < 0.001) compared to patients without such organ involvement. The PLR marker was substantially higher in patients exhibiting haematological disorders in the course of SLE (276.6 ± 226.4 vs 192.6 ± 133.5; *p* = 0.01). The results indicate that ELR and MLR are effective markers of SLE activity. The haematological indicators may predict SLE-dependent organ damage, particularly cutaneous, mucosal, arthritic, myositic, haematological and kidney involvement.

## Introduction

Systemic lupus erythematosus (SLE) is a chronic autoimmune disease that leads to inflammation of multiple tissues and organs. The constitutional symptoms of SLE predominantly involve damage to the skin, joints, kidneys, the central nervous system and bone marrow [1]. The diagnosis of the disease was based on the 2012 Systemic Lupus International Collaborating Clinics (SLICC) qualification criteria and the 2019 EULAR/ACR criteria [2, 3]. SLE treatment depends on the disease activity as well as the type of organs involved. In clinical practice, the assessment of disease activity in SLE is performed by means of disease activity scores Systemic Lupus Erythematosus Disease Activity Index 2000 (SLEDAI-2K) or additional testing for the complement components 3 and 4 (C3, C4) and anti-dsDNA titers [4]. The search for new markers of SLE activity is ongoing [5, 6]. In recent years, there has been a surge of interest in the role of haematological indicators in the assessment of SLE activity. Neutrophil-, basophil-, eosinophil-, monocyte- and platelet-to-lymphocyte ratios (NLR, BLR, ELR, MLR and PLR) have been found to indirectly reflect subclinical inflammation [7]. Their value for assessing or projecting the disease activity has been established in autoimmune diseases, such as primary Sjögren’s syndrome (pSS), psoriasis, systemic vasculitis, ulcerative colitis as well as in cancer and infectious diseases [8–15].

## Objectives

The objective of this study was to determine the usefulness of haematological indicators in the assessment of SLE activity and organ involvement.

## Patients and methods

A total of 136 patients with SLE (124 women and 12 men) aged 40.1 ± 14 years (22–74) hospitalised in the Clinic of Rheumatology and Connective Tissue Diseases at the Medical University of Lublin between 2012 and 2018 were enroled in the present retrospective study. All patients conformed with the 2012 SLICC criteria for SLE. Cutaneous and mucosal symptoms were present in 26 patients (19.1%), arthritis in 13 patients (9.5%), lupus nephritis in 23 patients (16.9%), haematological symptoms in 26 (19.1%) and vasculitis was manifested in 5 (3.7%). Two patients exhibited signs of damage to the nervous system and serositis. With respect to treatment, the patients received chloroquine/hydroxychloroquine (HQ/HCQ) monotherapy or HQ/HCQ therapy combined with low/medium doses of glucocorticoids (prednisone 6.3 mg/per day; 2.5–10 mg). Patients treated with high doses of glucocorticoids and strong immunosuppressants, overlap syndrome, infections, cancer, end-stage renal failure, diseases of the liver or the haematopoietic system were excluded from the study group.

The patients were evaluated for disease activity according to the SLEDAI-2K scale. In each patient, NLR, BLR, ELR, MLR and PLR indicators were determined (Table 1). The haematological markers were subsequently correlated with disease activity parameters, C-reactive protein (CRP) and erythrocyte sedimentation rate (ESR). The average values of the markers were compared across individual SLE-induced organ damage manifestations (Table 2).

**Table 1:** The haematological indicators and disease activity parameters in systemic lupus erythematosus patients

Haematological indicators and disease activity parameters; *n* = 136	Median value (min.–max.)
CRP (mg/dl) (range 0–5)	8.1 (0–107)
ESR (mm/h)	23.4 (0.12–109)
C3 (mg/dl) (range 85–160)	91.7 (11.4–151.8)
SLEDAI–2K	3.5 (2–22)
Anti–dsDNA (% positive)	101/136 (74%)
NLR	3.46 (0.6–22.2)
BLR	0.02 (0–0.4)
ELR	0.09 (0–1.3)
MLR	0.4 (0.1–9.9)
PLR	208.6 (18–1090)

*anti*-*dsDNA* anti-dsDNA antibodies, *C3* the complement component 3, *CRP* C-reactive protein, ESR erythrocyte sedimentation rate, *BLR* basophil-to-lymphocyte ratio, *ELR* eosinophil-to-lymphocyte ratio, *MLR* monocyte-to-lymphocyte ratio, *NRL* neutrophil-to-lymphocyte ratio, *PLR* platelet-to-lymphocyte ratio, *SLEDAI*-*2* *K* Systemic Lupus Erythematosus Disease Activity Index 2000

**Table 2:** A comparison of NLR, BLR, ELR, MLR and PLR concentrations in systemic lupus erythematosus patients in various manifestations of organ damage

Symptoms of SLE	NLR	BLR	ELR	MLR	PLR	*p*
Cutaneous and/or mucosal						
(+) *n* = 26	4.26 (0.8–22.2)*	0.025 (0–0.1)	0.1 (0–0.7)	0.51 (0.1–5.0)	236.5 (81–890.4)	*p* = 0.05*
(−) *n* = 110	3.27 (0.6–21.4)	0.022 (0–0.4)	0.09 (0–1.3)	0.39 (0.1–9.9)	202.08 (18–1090)	
Vasculitis						*p* < 0.001* *p* < 0.001** *p* < 0.001***
(+) *n* = 5	3.09 (1.2–4.4)	0.09 (0–0.4)*	0.4 (0–1,3)**	3.1 (0.1–9.9)***	199.68 (123.5–314.7)	
(−) *n* = 131	3.47 (0.6–22.2)	0.02 (0–0.1)	0.08 (0–0.3)	0.3 (0–1.5)	209.01 (18–1090)	
Arthritis and/or myositis						
(+) *n* = 13	2.76 (1.2–6.6)	0.04 (0–0.4)*	0.15 (0–1.3)	1.02 (0.1–9.9)**	215.9 (70.6–679.4)	*p* = 0.01*
(−) *n* = 123	3.53 (0.6–22.2)	0.02 (0–0.1)	0.09 (0–0.7)	0.35 (0.1–5.0)	207.9 (18–1090)	*p* = 0.01**
Nephritis						
(+) *n* = 23	5.45 (0.6–22.2)*	0.03 (0–0.1)	0.12 (0–0.7)	0.56 (0.1–5)	271.3 (41.8–890.5)**	*p* < 0.001* *p* = 0.03**
(−) *n* = 113	3.05 (0.9–13.6)	0.02 (0–0.4)	0.09 (0–1.3)	0.38 (0.1–9.9)	195.9 (18–890.5)	
Haematological						
(+) *n* = 26	4.1 (0.6–22.2)	0.02 (0–0.1)	0.07 (0–0.3)	0.3 (0.1–1.5)	276.6 (41.8–1090)*	*p* = 0.01*
(−) *n* = 110	3.3 (0.8–21.4)	0.02 (0–0.4)	0.1 (0–1.3)	0.4 (0.1–9.9)	192.6 (18–890.5)	
Anti-dsDNA						
(+) *n* = 101	3.7 (0.6–22.2)*	0.02 (0–0.1)	0.09 (0–0.7)	0.36 (0.1–5)	214.7 (41.8–890.5)	*p* = 0.03*
(−) *n* = 35	2.8 (1.1–9.6)	0.03 (0–0.4)	0.11 (0–1.3)	0.58 (0.1–9.9)	191.3 (18–1090)	

*anti*-*dsDNA* anti-dsDNA antibodies, *BLR* basophil-to-lymphocyte ratio, *ELR* eosinophil-to-lymphocyte ratio, *MLR* monocyte-to-lymphocyte ratio, *NRL* neutrophil-to-lymphocyte ratio, *PLR* platelet-to-lymphocyte ratio

*, **, *** indicates statistically significant differences between two values

### Statistical analysis

The analysis was carried out using STATISTICA 10 software. For numerical variables, descriptive statistics were calculated: median, minimum and maximum values. The Mann–Whitney U test was used for comparison of continuous variables in the two groups (with/without examined SLE symptoms). The Spearman rank correlation test was used to search for any relationships between variables. A *p* value < 0.05 was considered to be statistically significant.

## Results

In Table 1, the examined haematological indicators and disease activity in SLE patients are presented.

**Fig. 1:**
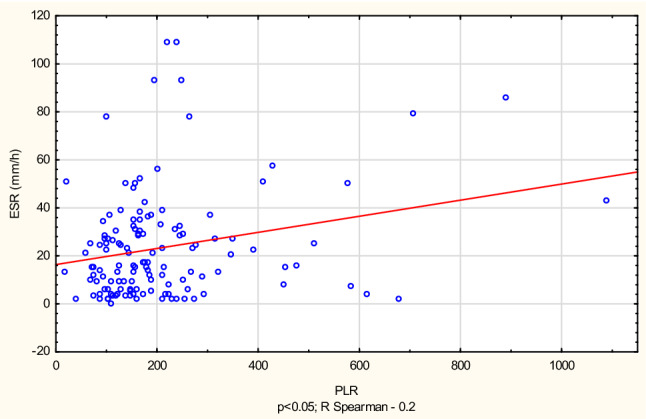
Correlation between erythrocyte sedimentation rate (ESR) and platelet-to-lymphocyte ratio (PLR)

**Fig. 2:**
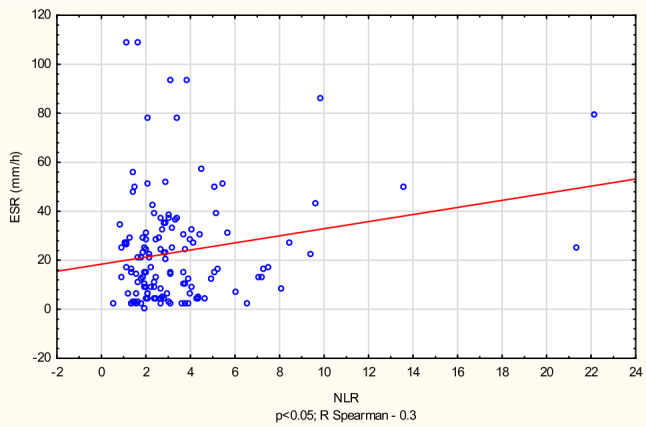
Correlation between erythrocyte sedimentation rate (ESR) and neutrophil-to-lymphocyte ratio (NLR)

The tests have revealed a positive correlation between ELR, MLR and the SLEDAI scale (*r* = 0.22 and *r* = 0.27, respectively). NLR, MLR and PLR ratios were significantly correlated with ESR (*r* = 0.24; *r* = 0.33; *r* = 0.2, respectively) (Figs. 1 and 2) and PLR with CRP (*r* = 0.2).

Table 2 compares the average concentrations of haematological indicators: NLR, BLR, ELR, MLR and PLR in patients with SLE with various manifestations of organ disease. The values of these indicators were not compared in patients presenting serositis and neurological symptoms due to the inadequate number of patients (2 patients) manifesting the symptoms. Considerably higher NLR values were found in patients with cutaneous and/or mucosal symptoms and with kidney involvement, compared to patients without such involvement. BLR and MLR were significantly higher in patients manifesting symptoms of vasculitis, arthritis and/or myositis, whereas elevated ELR ratios were observed in patients with vasculitis compared to patients without such organ involvement. The PLR marker was substantially higher in patients exhibiting haematological disorders in the course of SLE.

## Discussion

In recent years, there has been a growing emphasis on early diagnosis of SLE exacerbation and monitoring of SLE activity. Simultaneously, concentrated efforts have been made to search for markers capable of prognosing SLE exacerbation in the preclinical period, which could, furthermore, indicate signs of exacerbation in a given organ. A considerable literature has provided evidence for the efficacy of haematological indicators, i.e. NLR, BLR, ELR, MLR and PLR in monitoring the activity of a number of inflammatory joint diseases, including SLE, rheumatoid arthritis (RA), pSS or systemic sclerosis. What is more, NLR has been proved to successfully predict the development of lupus nephritis (LN).

From the results of our study, it can be seen that ELR and MLR significantly correlated with the SLEDAI scale, whereas NLR and PLR exhibited no relationship with the disease activity markers. There was a significant positive correlation between NLR/MLR/PLR indicators and ESR/CRP values. With respect to organ involvement, significantly higher NLR was observed in patients with cutaneous and/or mucosal symptoms and kidney damage. Moreover, NLR was also elevated in patients with anti-dsDNA antibodies. BLR and MLR were significantly higher in patients manifesting symptoms of vasculitis, arthritis and/or myositis, while ELR—in patients with vasculitis. Significantly higher PLR rates were recorded in patients with haematological manifestations of SLE. ELR and BLR demonstrated a good capacity for assessing SLE activity. Considering the prognosis of organ involvement, there were NLR, MLR, BLR and ELR that showed good performance in the assessment of cutaneous lesion, NLR and PLR in the prediction of renal damage, whereas BLR and MLR showed good correlation with arthritic manifestations and PLR with haematological symptoms.

Waffa et al. observed a significant correlation between NLR/PLR and the SLEDAI scale and C4. Moreover, in the course of their study, NLR was found to be a good marker of renal function parameters, proteinuria, anti-dsDNA and histopathological changes in kidney biopsy. Both NLR and PLR exhibited sufficient accuracy in the prediction of kidney damage in SLE. What is more, the haematological markers correlated well with the inflammation markers ESR and CRP [9]. Similar findings were presented by Qin et al. [16]. In contrast to earlier findings, in our study, the relationship was observed between NLR and ESR.

Wu et al. reported elevated levels of NLR and PLR in patients with active SLE. The neutrophil-to-lymphocyte ratio was significantly higher in patients with kidney involvement and was, furthermore, an accurate marker of SLE exacerbation [17]. A similar relationship in SLE patients has been confirmed in earlier studies: Soliman et al., Li et al. and Ayna et al. [18–20]. While confirming the predictive value of NLR in assessing SLE activity, Yu et al. determined a new indicator—NC3R—neutrophil-to-C3 ratio [21]. In a far more extensive study, Yang et al. examined 1139 patients presenting a range of inflammatory arthritic diseases (SLE, RA, pSS, dermatomyositis, polymyositis, mixed connective tissue disease, rheumatic polymyalgia and ankylosing spondylitis) and osteoarthritis (OA). From their findings, it can be seen that notably higher NLR and MLR ratios were prevalent in patients with inflammatory arthritic diseases. In the majority of patients, significantly lower BLR was observed. Compared with other inflammatory arthritic diseases, lower ELR was recorded in patients with SLE [22]. Ma et al. and Wang et al. performed meta-analysis to investigate the relationship between NLR, PLR and SLE. Fourteen studies with 1246 SLE patients (Ma et al.) and 1781 SLE patients (Wang et al.) were included in this meta-analysis. The meta-analysis demonstrated elevated levels of NLR and PLR in patients with active SLE (Ma et al.) and positive clinical value of NRL for diagnosing SLE, active SLE or LN (Wang et al.) [23, 24].

Despite SLE activity scores being widely available for clinicians, haematological indicators constitute a considerably faster and more easily accessible biomarker of the disease activity. In the available literature, there is only one study that assesses the predictive value of all five haematological indicators (NLR, MLR, BLR, ELR and PLR) in monitoring SLE activity. In earlier works, however, the markers were not measured in correlation with organ manifestations of SLE, with the exception of patients with lupus nephritis. Although there are limitations due to a relatively small group of patients, the retrospective analysis approach or short-term observation, we are inclined to believe that these cost-effective and widely available markers could constitute a positive addition to a daily clinical practice involving the assessment of SLE activity.

In conclusion, the haematological indicators may predict SLE-dependent organ damage, particularly cutaneous, mucosal, arthritic, myositic, haematological and kidney involvement. Future studies should account for a comparative analysis of the indicators in question in various systemic diseases.
